# Analysis of aqueous humor concentrations of cytokines in retinoblastoma

**DOI:** 10.1371/journal.pone.0177337

**Published:** 2017-05-09

**Authors:** Yong Cheng, Shufeng Zheng, Chung-Ting Pan, Mengke Yuan, Libin Chang, Yuou Yao, Mingwei Zhao, Jianhong Liang

**Affiliations:** 1 Department of Ophthalmology, People’s Hospital, Peking University, Beijing, China; 2 Key Laboratory of Vision Loss and Restoration, Ministry of Education, Beijing, China; 3 Beijing Key Laboratory of Diagnosis and Therapy of Retinal and Choroid Diseases, Beijing, China; 4 Eye Hospital, Tradition Chinese Medicine Hospital of Yulin, Shaanxi, China; 5 Department of Ophthalmology, Peking University International Hospital, Beijing, China; 6 Department of Ophthalmology, Beijing Shunyi Airport Hospital, Beijing, China; 7 Department of Ophthalmology, Beijing Jingmei Group General Hospital, Beijing, China; Medizinische Universitat Graz, AUSTRIA

## Abstract

To investigate the components of the aqueous humor (AH) in patients with retinoblastoma (RB). We collected 0.1 ml AH of 35 children with RB and 20 patients with congenital cataracts as controls. Multiplex enzyme-linked immunosorbent assays (ELISAs) and Luminex xMAP technology were used to assess 45 cytokines/chemokines, matrix metalloproteinases (MMPs), and acute-phase proteins in the identification cohort. The concentrations of IL-6, IL-7, IL-8, IFN-γ, PIGF-1, VEGF-A, β-NGF, HGF, EGF and FGF-2 were significantly higher in the AH of patients with RB than those in the control group (P<0.05). The study showed that the higher levels of IP-10, IL-6, IL-7, IL-8, IFN-γ, PIGF-1, VEGF-A, β-NGF, HGF, EGF and FGF-2 in AH may be associated with RB. Our findings may facilitate a better understanding of the molecular pathways of tumors and solid molecular targets for new strategies for therapy and the earlier diagnosis of RB.

## Introduction

With an incidence of 7,000–8,000 new cases per year worldwide, retinoblastoma (RB) is the most common primary intraocular malignant tumor in children [1]. Nearly 80% of cases of retinoblastoma are diagnosed before 3 years of age, while only extremely rare cases of retinoblastoma are confirmed after 6 years old [2]. Leukocoria is the major presentation in children with RB [3]. Funduscopy, ultrasonography, computed tomography (CT), magnetic resonance imaging (MRI) and histology help ophthalmologists to make a diagnosis of RB [2,4]. Although modern technology and early diagnosis has led to a favorable improvement in prognosis, RB is still life-threatening in cases of late diagnosis. Therefore, earlier diagnoses are essential for curing RB.

The aqueous humor (AH) is an intraocular fluid containing various proteins and cytokines with changes in concentration under disease status. Dr. Theodora Hadjistilianou and colleagues found that the AH protein concentration was significantly higher in RB patients. However, they did not characterize and analyze the precise proteins [5]. No other paper has illustrated the specific protein composition until now. The identification of proteins and cytokines in AH may have a potential role in the understanding of pathogenesis and may inspire some novel concepts for therapeutic innovation.

This study was performed to assess the expression of cytokines in AH samples of RB patients for further diagnosis and therapy.

## Patients and methods

This was a prospective case series. The study was approved by the Clinical Institutional Review Board of Peking University People’s Hospital and was conducted in concordance with the tenets of the Declaration of Helsinki. Thirty-five samples of AH of patients who were diagnosed with RB in clinic at Peking University People’s Hospital from September 2014 to March 2015 were reviewed for entry. The control (CTR) group consisted of 20 AH samples of patients undergoing routine cataract surgery at the Ophthalmology Department of Peking University People’s Hospital. Written informed consent was obtained from the guardian of each participant. The inclusion criterion in the RB group was patients diagnosed with group D or group E retinoblastoma according to the International Classification of Retinoblastoma (ICRB) [6]. All RB patients underwent enucleation of the affected eyes without any previous treatment. Patients and controls with a history of ocular surgery or trauma or other disorders were excluded because this may have confounded the results.

Approximately 0.1 ml AH was collected under a binocular microscope by paracentesis of the anterior chamber. This procedure was performed immediately after the ocular enucleation in the RB group and a stab peripheral corneal incision in the CTR group to prevent the intraocular tumor from becoming extraocular or even metastatic [2]. Samples were stored at -80°C immediately until laboratory analysis. A 45-plex Human Cytokine/Chemokine/Growth Factor Elisa Kit (eBioscience, Procarta) was used to determine the AH levels of cytokines, chemokines and growth factors (CCGFs), including brain-derived neurotrophic factor (BDNF), epidermal growth factor (EGF), eotaxin/CCL11, basic fibroblast growth factor (FGF-2), granulocyte-macrophage colony stimulating factor (GM-CSF), growth-regulated oncogene α (GROα/CXCL1), hepatocyte growth factor (HGF), interferon gamma (IFN-γ), IFNα, interleukin (IL)-1RA, IL-1β, IL-1α, IL-2, IL-4, IL-5, IL-6, IL-7, IL-8/CXCL8, IL-9, IL-10, IL-12 p70, IL-13, IL-15, IL-17A, IL-18, IL-21, IL-22, IL-23, IL-27, IL-31, interferon-induced protein10 (IP-10/CXCL10)), leukemia inhibitory factor (LIF), monocyte chemotactic protein 1 (MCP-1/CCL2), macrophage inflammatory protein-1α (MIP-1α/CCL3), MIP-1β/CCL4, β-nerve growth factor (β-NGF), platelet-derived growth factor-BB (PDGF-BB), placental growth factor (PLGF), regulated upon activation normal T cell (RANTES/CCL5), stem cell factor (SCF), stromal cell derived factor-1α (SDF1α/CXCL12), tumor necrosis factor-α(TNF-α), TNF-β/lymphotoxin α (LTA), placental growth factor-1(PIGF-1), vascular endothelial growth factor A (VEGF-A) and VEGF-D. The detailed procedure for the assessment of AH cytokines using Luminex xMAP technology with multi-analyte profiling beads was described in previous reports [7,8]. A significance level of 95% (P < 0.05) was used for all statistical analyses.

### Statistical methods

Data were analyzed using a commercially available statistical software program, SPSS (SPSS for Windows, version 22; IBM/SPSS, Chicago, IL). Quantitative variables were presented as the means ± standard deviation. Independent-samples t test was used to compare the data between RB and control values.

## Results

The study population included 35 eyes of 35 RB patients and 20 eyes of 20 controls who underwent surgery from 2014–2015. Demographic characteristics of the patients and controls are presented in Table 1. There were no statistically significant differences between the two groups in terms of gender and age (P> 0.05). The study group included 24 (68.57%) group D RB patients and 11 (31.43%) group E RB patients.

**Table 1:** Demographics of the study population.

Characteristic		RB patients	Controls	P Value
		(n = 35)	(n = 20)	
**Gender**	**Male**	**22 (62.86%)**	**12 (60.00%)**	** **
	**Female**	**13 (37.14%)**	**8(40.00%)**	**>0.05**
**Age(months)**	**mean(±SD)**	**18.45 ± 6.36**	**15.36 ± 6.62**	
	**Range**	**11~32**	**8~28**	**>0.05**
**Number Classification as RB**	**Group D**	**24(68.57%)**	**-**	**-**
	**Group E**	**11(31.43%)**	**-**	**-**

RB, retinoblastoma

A total of 55 aqueous humor samples from 35 RB patients and 20 controls were collected. Our analyses of the aqueous cytokine levels showed dramatically different concentrations of 10 cytokines except EGF between the RB group and CTR group (Table 2). Among the 10 cytokines, the concentrations of IL-6, IL-7, IL-8, IFN-γ, PIGF-1, VEGF-A, β-NGF, HGF, EGF and FGF-2 were significantly higher in the AH of patients with RB than the control group (P<0.05). IP-10 showed a lower concentration in the RB group than the CTR group.

**Table 2:** Aqueous humor levels of cytokines in eyes with RB and cataracts.

Cytokines	RB group	(n = 35)	CTR group	(n = 20)	F value	P value
	AH (pg/ml)	Standard error	AH (pg/ml)	Standard error	(t test)	(t test)
**IP-10**	**1.27**	**1.14**	**24.93**	**1.13**	**9.124**	**0.004**
**IL-6**	**37.58**	**8.06**	**4.86**	**3.63**	**6.464**	**0.015**
**IL-7**	**7.89**	**2.49**	**4.75**	**2.27**	**0.002**	**0.016**
**IL-8**	**87.23**	**9.02**	**17.9**	**1.92**	**2.215**	**0.045**
**IFN-γ**	**5.28**	**1.96**	**0.8**	**0.13**	**1.088**	**0.003**
**PIGF-1**	**16.27**	**2.95**	**1**	**0.85**	**4.695**	**0.039**
**VEGF-A**	**14.91**	**5.6**	**2.88**	**1.29**	**6.828**	**0.012**
**HGF**	**8.21**	**2.45**	**2.43**	**3.29**	**7.112**	**0.011**
**β-NGF**	**19.56**	**2.4**	**5.97**	**3.16**	**5.057**	**0.03**
**EGF**	**3.97**	**1.07**	**0.66**	**0.22**	**4.138**	**0.05**
**FGF-2**	**29.94**	**8.62**	**18.41**	**7.58**	**1.082**	**0.037**

IL, interleukin, IP-10, interferon-induced protein10, PIGF-1, placenta growth factor 1, IFN-γ, interferon gamma, HGF, hepatocyte growth factor, β-NGF, beta-nerve growth factor, EGF, epidermal growth factor, FGF-2, fibroblast factor 2, VEGF-A, vascular endothelial growth factor A, CTR, control

## Discussion

Retinoblastoma (RB) is the most common intraocular malignancy in children. Although modern technology and early diagnosis have reduced mortality to less than 5% in Europe and the USA, the mortality rate in Africa is still 70% [1]. Although current therapeutic strategies have led to a dramatic improvement in individual prognosis, RB is still life-threatening when left untreated or in cases of late diagnosis. It is really a concern in less-developed countries. The functions of AH include maintaining intraocular pressure, supplying nutrients for non-vascularized ocular tissues, and removing the products of metabolism and ascorbic acid transportation to protect against oxidation [9]. The expression profile of different proteins in AH changes in different diseases [10,11]. Accordingly, the identification of AH proteins may help to recognize their potential character in pathogenesis. Some researchers have reported that by using multidimensional protein identification technology, the proteomic contains of AH could be analyzed [12,13]. Proteins associated with inhibition and binding of proteolytic activity or related to functions of immunoregulation, autoxidation and transportation were identified [12,13]. In our study, we discovered that intraocular concentrations of IL-6, IL-7, IL-8, PIGF-1, IFN-γ, HGF, β-NGF, FGF-2 and VEGF-A were significantly higher in RB patients than in the CTR group, while IP-10 showed the opposite. To the best of our knowledge, this is the first attempt to research human AH cytokines in RB patients.

In our study, the level of VEGF-A (known as VEGF) in AH was significantly high, which has never been reported before. In previous studies, the VEGF mRNA in the paraffin sections of tumor mass was over-expressed [14], and the level of the VEGF-A in vitreous fluids was significantly high [15]. Focal hypoxia may stimulate the production of VEGF in retinoblastomas, and VEGF production may contribute to tumor growth [14]. VEGF is an angiogenic factor essential for both physiological and pathological angiogenesis, inducing the proliferation and migration of endothelial cells, and in combination with PIGF into heterodimers [16]. VEGF-A and PIGF belong to the VEGF family, which mediate angiogenesis and lymphangiogenesis in tumors [17]. VEGF-A may be modulated by PIGF through the expression of VEGFR-1 on endothelial cells [17]. In addition to having pro-angiogenic effects, VEGF also has direct effects on tumor cell survival, migration and invasion according to the stage and process of the tumor and can also suppress immune function [16]. The growth of RB requires large-scale vascularization. Therefore, the anti-VEGF strategy should theoretically be a prospective therapy in treating RB patients. Previous studies have described the anti-tumor effects of anti-VEGF [18–21]. It has been reported that PIGF takes part in cancer as a paracrine and autocrine factor, promoting proliferation and as a stimulator of angiogenesis of tumor cells [17], and this might explain the high concentration of PIGF in our study. The EGF binds to epidermal growth factor receptor (EGFR) to induce the expression of VEGF-A by triggering downstream Ras-MAPK, PI3K-Akt, and STAT signaling pathways, which play important roles in tumor growth and dissemination [22–24]. Therefore, the blockage of Ras-MAPK/PI3K-Akt/STAT signaling pathways might be a future strategy for RB. The level of EGF was higher in the RB group than in the CTR group, but it did not reach statistical significance in our study. This may be associated with the small sample size. Anti-VEGF, anti-PIGF, blockage of signaling pathways or combination therapies for RB require further investigation.

It has been reported that inflammation leads to cancer development by producing several inflammatory mediators [25]. In our study, the levels of IL-6, IL-7, IL-8, and IFN-γ were significantly higher in the RB group, indicating a continuous inflammatory condition in RB patients. However, the aqueous level of IP-10 showed a significantly lower concentration in the RB group than in the CTR group, which was inconsistent with previous studies [26,27]. IL-6, IL-7, IL-8, IFN-γ and IP-10 were reported to be associated with inflammation in diabetic retinopathy, branch and central retinal vein occlusion and choroidal neovascularization [8,28–32]. Previous studies reported that IL-6 directly inactivates retinoblastoma protein (pRb) [33,34]. Moreover, IL-6 indirectly inactivates the phosphorylation of PRb [35]. PRb is a tumor suppressorprotein that is dysfunctional in many cancers [33,34]. Patients with cancers tend to have an immunosuppressive status with increased T cell counts. It has been reported that elevated IL-7 expression in prostate cancer is associated with poor prognosis. The IL-7/IL-7R axis is related to cell invasion and migration, and blocking the IL-7/IL-7R axis may treat prostate cancer [36]. IL-8 and its receptor show a correlation with the increased risk, progression and invasion of breast cancer [37,38]. In our study, the level of IL-8 increased in RB. It has been proposed that blocking IL-6, IL-7 and IL-8 functions in combination with drug therapy would potentially treat RB. IP-10 is significantly increased in patients with tuberculosis(TB), coronary atherosclerosis, systemic lupus erythematosus (SLE)and thyroid cancer (TC) [39–42]. However, IP-10 is expressed at a low level in our study. We hypothesized that there was a delay in IP-10 expression or a peak concentration in IP-10 expression, or other unknown factors and pathways affected the concentration of IP-10.

The three remaining cytokines are FGF2, β-NGF and HGF. FGF2, HGF and EGF are associated with the angiogenesis, growth, proliferation and differentiation of numerous cell types, including certain tumor cells [43–45]. Our findings showed that the levels of FGF2, β-NGF and HGF were strikingly higher in RB patients consistent with Cebulla CM’s study [43]. All these factors play a part in vascularization and cell proliferation and produce a consistent outcome according to increased levels of VEGF and PIGF, as we predicted [43–46]. These cytokines are important cofactors in terms of nerve growth, vascularization, invasiveness and cell healing, indicating a complicated process during tumor development. Future studies are needed to evaluate whether changes of the FGF2, HGF or β-NGF pathways in RB could lead to future therapeutic targets for the disease.

In summary, our study showed that several cytokines and factors in the aqueous humor were associated with RB. Inflammation and angiogenesis are hallmarks of RB. This is the first analysis to investigate the aqueous humor cytokines in RB patients. Analysis of AH RB-related cytokines will help scientists understand the tumor molecular pathways and shed new light onto this ocular disease. This may lead to the discovery of novel molecular targets for an early diagnosis and new cancer therapy strategies.

## Supporting information

S1 Table Aqueous humor levels of cytokines without significant difference in eyes with RB and cataracts. Still other AH concentrations of proteins were so few that they cannot be tested, so the data of which were not included in S1 Table.
(DOCX)
