# Supplementary material for: Analysis of aqueous humor concentrations of cytokines in retinoblastoma
Source: PLoS One. 2017 May 9;12(5):e0177337. doi: 10.1371/journal.pone.0177337 (PMC5423669; doi:10.1371/journal.pone.0177337)
Supplement: S1 Table — Still other AH concentrations of proteins were so few that they cannot be tested, so the data of which were not included in S1 Table. (DOCX) [file pone.0177337.s001.docx]

**Supporting information**

**S1 Table. Aqueous humor levels of cytokines without significant difference in eyes with RB and cataracts.** Still other AH concentrations of proteins were so few that they cannot be tested, so the data of which were not included in S1 Table.
